# Supplementary material for: Determining novel candidate anti-hepatocellular carcinoma drugs using interaction networks and molecular docking between drug targets and natural compounds of SiNiSan
Source: PeerJ. 2021 Feb 16;9:e10745. doi: 10.7717/peerj.10745 (PMC7894118; doi:10.7717/peerj.10745)
Supplement: Supplemental Information 5 [file peerj-09-10745-s005.html]

Report for 1 1593766509348 [GSEA]

| GS  follow link to MSigDB | GS DETAILS | SIZE | ES | NES | NOM p-val | FDR q-val | FWER p-val | RANK AT MAX | LEADING EDGE || 1 | PID\_PLK1\_PATHWAY | Details ... | 44 | 0.85 | 1.80 | 0.000 | 0.033 | 0.067 | 1429 | tags=48%, list=7%, signal=51% |
| 2 | PID\_AURORA\_B\_PATHWAY | Details ... | 37 | 0.84 | 1.75 | 0.002 | 0.037 | 0.117 | 558 | tags=43%, list=3%, signal=44% |
| 3 | PID\_FANCONI\_PATHWAY | Details ... | 41 | 0.72 | 1.72 | 0.008 | 0.039 | 0.150 | 4035 | tags=66%, list=19%, signal=81% |
| 4 | PID\_FOXM1\_PATHWAY | Details ... | 39 | 0.68 | 1.71 | 0.006 | 0.034 | 0.163 | 1516 | tags=46%, list=7%, signal=50% |
| 5 | PID\_ATR\_PATHWAY | Details ... | 38 | 0.78 | 1.70 | 0.004 | 0.030 | 0.178 | 1281 | tags=50%, list=6%, signal=53% |
| 6 | PID\_DELTA\_NP63\_PATHWAY | Details ... | 43 | 0.46 | 1.63 | 0.004 | 0.056 | 0.290 | 618 | tags=16%, list=3%, signal=17% |
| 7 | PID\_HIF1A\_PATHWAY | Details ... | 16 | 0.67 | 1.61 | 0.034 | 0.064 | 0.342 | 4015 | tags=44%, list=19%, signal=54% |
| 8 | PID\_P73PATHWAY | Details ... | 76 | 0.49 | 1.57 | 0.002 | 0.082 | 0.416 | 1281 | tags=20%, list=6%, signal=21% |
| 9 | PID\_BARD1\_PATHWAY | Details ... | 28 | 0.64 | 1.56 | 0.038 | 0.079 | 0.431 | 4035 | tags=54%, list=19%, signal=66% |
| 10 | PID\_E2F\_PATHWAY | Details ... | 72 | 0.57 | 1.55 | 0.051 | 0.079 | 0.452 | 1993 | tags=35%, list=9%, signal=38% |
| 11 | PID\_ATM\_PATHWAY | Details ... | 30 | 0.59 | 1.50 | 0.077 | 0.112 | 0.554 | 3526 | tags=40%, list=16%, signal=48% |
| 12 | PID\_AURORA\_A\_PATHWAY | Details ... | 31 | 0.66 | 1.47 | 0.053 | 0.125 | 0.589 | 1843 | tags=32%, list=9%, signal=35% |
| 13 | PID\_WNT\_SIGNALING\_PATHWAY | Details ... | 25 | 0.45 | 1.37 | 0.071 | 0.245 | 0.744 | 400 | tags=12%, list=2%, signal=12% |
| 14 | PID\_MYC\_ACTIV\_PATHWAY | Details ... | 74 | 0.48 | 1.27 | 0.244 | 0.422 | 0.884 | 1593 | tags=20%, list=7%, signal=22% |
| 15 | PID\_P38\_MK2\_PATHWAY | Details ... | 21 | 0.49 | 1.24 | 0.277 | 0.443 | 0.903 | 1299 | tags=14%, list=6%, signal=15% |
| 16 | PID\_RB\_1PATHWAY | Details ... | 63 | 0.39 | 1.22 | 0.267 | 0.472 | 0.930 | 3992 | tags=29%, list=18%, signal=35% |
| 17 | PID\_CDC42\_REG\_PATHWAY | Details ... | 30 | 0.36 | 1.16 | 0.290 | 0.566 | 0.964 | 697 | tags=7%, list=3%, signal=7% |
| 18 | PID\_DNA\_PK\_PATHWAY | Details ... | 15 | 0.38 | 1.12 | 0.339 | 0.626 | 0.978 | 3526 | tags=33%, list=16%, signal=40% |
| 19 | PID\_LIS1\_PATHWAY | Details ... | 28 | 0.36 | 1.12 | 0.323 | 0.594 | 0.978 | 5746 | tags=39%, list=27%, signal=53% |
| 20 | PID\_SYNDECAN\_2\_PATHWAY | Details ... | 31 | 0.35 | 1.11 | 0.341 | 0.576 | 0.979 | 4317 | tags=23%, list=20%, signal=28% |
| 21 | PID\_P53\_REGULATION\_PATHWAY |  | 54 | 0.39 | 1.10 | 0.433 | 0.574 | 0.982 | 6338 | tags=41%, list=29%, signal=57% |
| 22 | PID\_INTEGRIN\_CS\_PATHWAY |  | 25 | 0.31 | 1.03 | 0.418 | 0.685 | 0.995 | 3269 | tags=24%, list=15%, signal=28% |
| 23 | PID\_P53\_DOWNSTREAM\_PATHWAY |  | 131 | 0.28 | 1.00 | 0.462 | 0.709 | 0.999 | 3032 | tags=19%, list=14%, signal=22% |
| 24 | PID\_WNT\_NONCANONICAL\_PATHWAY |  | 32 | 0.38 | 0.99 | 0.520 | 0.703 | 0.999 | 5904 | tags=34%, list=27%, signal=47% |
| 25 | PID\_RETINOIC\_ACID\_PATHWAY |  | 30 | 0.32 | 0.91 | 0.658 | 0.822 | 0.999 | 35 | tags=3%, list=0%, signal=3% |
| 26 | PID\_SYNDECAN\_1\_PATHWAY |  | 46 | 0.30 | 0.83 | 0.688 | 0.945 | 1.000 | 2613 | tags=20%, list=12%, signal=22% |
| 27 | PID\_INSULIN\_GLUCOSE\_PATHWAY |  | 26 | 0.33 | 0.82 | 0.690 | 0.922 | 1.000 | 5493 | tags=35%, list=25%, signal=46% |
| 28 | PID\_RAS\_PATHWAY |  | 29 | 0.26 | 0.81 | 0.699 | 0.913 | 1.000 | 4108 | tags=21%, list=19%, signal=25% |
| 29 | PID\_MYC\_PATHWAY |  | 24 | 0.31 | 0.80 | 0.692 | 0.899 | 1.000 | 2804 | tags=21%, list=13%, signal=24% |
| 30 | PID\_PRL\_SIGNALING\_EVENTS\_PATHWAY |  | 23 | 0.35 | 0.79 | 0.743 | 0.889 | 1.000 | 3877 | tags=26%, list=18%, signal=32% |
| 31 | PID\_ERB\_GENOMIC\_PATHWAY |  | 15 | 0.31 | 0.75 | 0.796 | 0.928 | 1.000 | 2619 | tags=20%, list=12%, signal=23% |
| 32 | PID\_ERBB1\_RECEPTOR\_PROXIMAL\_PATHWAY |  | 35 | 0.26 | 0.71 | 0.754 | 0.966 | 1.000 | 4919 | tags=26%, list=23%, signal=33% |
| 33 | PID\_RAC1\_PATHWAY |  | 53 | 0.26 | 0.67 | 0.806 | 0.996 | 1.000 | 957 | tags=6%, list=4%, signal=6% |
| 34 | PID\_CONE\_PATHWAY |  | 22 | 0.32 | 0.67 | 0.898 | 0.966 | 1.000 | 6214 | tags=41%, list=29%, signal=57% |
| 35 | PID\_RHODOPSIN\_PATHWAY |  | 22 | 0.24 | 0.67 | 0.926 | 0.944 | 1.000 | 2584 | tags=14%, list=12%, signal=15% |
| 36 | PID\_MTOR\_4PATHWAY |  | 67 | 0.25 | 0.66 | 0.833 | 0.924 | 1.000 | 2431 | tags=12%, list=11%, signal=13% |
| 37 | PID\_ILK\_PATHWAY |  | 44 | 0.27 | 0.66 | 0.836 | 0.905 | 1.000 | 1409 | tags=9%, list=7%, signal=10% |
| 38 | PID\_LKB1\_PATHWAY |  | 46 | 0.25 | 0.64 | 0.861 | 0.900 | 1.000 | 741 | tags=7%, list=3%, signal=7% |
| 39 | PID\_A6B1\_A6B4\_INTEGRIN\_PATHWAY |  | 46 | 0.21 | 0.63 | 0.915 | 0.895 | 1.000 | 2431 | tags=11%, list=11%, signal=12% |
| 40 | PID\_SMAD2\_3PATHWAY |  | 16 | 0.31 | 0.62 | 0.854 | 0.878 | 1.000 | 303 | tags=6%, list=1%, signal=6% |
| 41 | PID\_WNT\_CANONICAL\_PATHWAY |  | 19 | 0.21 | 0.58 | 0.885 | 0.893 | 1.000 | 6185 | tags=32%, list=29%, signal=44% |
Table: Gene sets enriched in phenotype **1 (16 samples)**[plain text format]****

  
