# Supplementary material for: Determining novel candidate anti-hepatocellular carcinoma drugs using interaction networks and molecular docking between drug targets and natural compounds of SiNiSan
Source: PeerJ. 2021 Feb 16;9:e10745. doi: 10.7717/peerj.10745 (PMC7894118; doi:10.7717/peerj.10745)
Supplement: Supplemental Information 6 [file peerj-09-10745-s006.html]

Report for 1 1593745746507 [GSEA]

| GS  follow link to MSigDB | GS DETAILS | SIZE | ES | NES | NOM p-val | FDR q-val | FWER p-val | RANK AT MAX | LEADING EDGE || 1 | PID\_ATR\_PATHWAY | Details ... | 39 | 0.75 | 2.01 | 0.000 | 0.000 | 0.000 | 6026 | tags=74%, list=11%, signal=83% |
| 2 | PID\_FANCONI\_PATHWAY | Details ... | 47 | 0.70 | 1.95 | 0.000 | 0.000 | 0.000 | 13278 | tags=85%, list=23%, signal=111% |
| 3 | PID\_AURORA\_B\_PATHWAY | Details ... | 39 | 0.71 | 1.92 | 0.000 | 0.000 | 0.000 | 3587 | tags=56%, list=6%, signal=60% |
| 4 | PID\_PLK1\_PATHWAY | Details ... | 46 | 0.69 | 1.92 | 0.000 | 0.000 | 0.000 | 5359 | tags=63%, list=9%, signal=70% |
| 5 | PID\_ATM\_PATHWAY | Details ... | 34 | 0.71 | 1.88 | 0.000 | 0.000 | 0.001 | 14484 | tags=91%, list=26%, signal=123% |
| 6 | PID\_LKB1\_PATHWAY | Details ... | 47 | 0.67 | 1.85 | 0.000 | 0.000 | 0.001 | 8107 | tags=64%, list=14%, signal=74% |
| 7 | PID\_P53\_REGULATION\_PATHWAY | Details ... | 59 | 0.65 | 1.84 | 0.000 | 0.000 | 0.001 | 15827 | tags=78%, list=28%, signal=108% |
| 8 | PID\_MTOR\_4PATHWAY | Details ... | 69 | 0.64 | 1.83 | 0.000 | 0.000 | 0.001 | 12355 | tags=67%, list=22%, signal=85% |
| 9 | PID\_ARF\_3PATHWAY | Details ... | 19 | 0.74 | 1.80 | 0.000 | 0.000 | 0.001 | 7347 | tags=79%, list=13%, signal=91% |
| 10 | PID\_MYC\_PATHWAY | Details ... | 25 | 0.71 | 1.80 | 0.000 | 0.000 | 0.003 | 6909 | tags=68%, list=12%, signal=77% |
| 11 | PID\_E2F\_PATHWAY | Details ... | 73 | 0.62 | 1.79 | 0.000 | 0.000 | 0.003 | 10700 | tags=60%, list=19%, signal=74% |
| 12 | PID\_FOXM1\_PATHWAY | Details ... | 40 | 0.64 | 1.76 | 0.000 | 0.000 | 0.005 | 6155 | tags=58%, list=11%, signal=64% |
| 13 | PID\_DNA\_PK\_PATHWAY | Details ... | 16 | 0.74 | 1.75 | 0.001 | 0.000 | 0.007 | 10820 | tags=75%, list=19%, signal=93% |
| 14 | PID\_AURORA\_A\_PATHWAY | Details ... | 31 | 0.67 | 1.75 | 0.000 | 0.001 | 0.008 | 7929 | tags=58%, list=14%, signal=68% |
| 15 | PID\_P38\_MK2\_PATHWAY | Details ... | 21 | 0.70 | 1.75 | 0.000 | 0.001 | 0.009 | 12189 | tags=81%, list=22%, signal=103% |
| 16 | PID\_BARD1\_PATHWAY | Details ... | 29 | 0.66 | 1.73 | 0.001 | 0.001 | 0.015 | 12289 | tags=72%, list=22%, signal=92% |
| 17 | PID\_HIF1A\_PATHWAY | Details ... | 19 | 0.69 | 1.69 | 0.000 | 0.002 | 0.041 | 14459 | tags=84%, list=26%, signal=113% |
| 18 | PID\_IGF1\_PATHWAY | Details ... | 29 | 0.63 | 1.66 | 0.000 | 0.003 | 0.061 | 11704 | tags=62%, list=21%, signal=78% |
| 19 | PID\_HDAC\_CLASSI\_PATHWAY | Details ... | 66 | 0.59 | 1.66 | 0.000 | 0.003 | 0.064 | 11802 | tags=56%, list=21%, signal=71% |
| 20 | PID\_SMAD2\_3PATHWAY | Details ... | 16 | 0.69 | 1.65 | 0.002 | 0.004 | 0.075 | 9486 | tags=69%, list=17%, signal=83% |
| 21 | PID\_ARF6\_DOWNSTREAM\_PATHWAY |  | 15 | 0.69 | 1.63 | 0.003 | 0.005 | 0.113 | 8518 | tags=60%, list=15%, signal=71% |
| 22 | PID\_P73PATHWAY |  | 79 | 0.56 | 1.62 | 0.000 | 0.006 | 0.122 | 8934 | tags=48%, list=16%, signal=57% |
| 23 | PID\_CASPASE\_PATHWAY |  | 51 | 0.57 | 1.56 | 0.000 | 0.014 | 0.304 | 17718 | tags=63%, list=31%, signal=91% |
| 24 | PID\_HEDGEHOG\_GLI\_PATHWAY |  | 48 | 0.56 | 1.56 | 0.000 | 0.014 | 0.304 | 14279 | tags=63%, list=25%, signal=84% |
| 25 | PID\_PI3KCI\_PATHWAY |  | 48 | 0.56 | 1.55 | 0.002 | 0.017 | 0.365 | 8072 | tags=38%, list=14%, signal=44% |
| 26 | PID\_MYC\_ACTIV\_PATHWAY |  | 79 | 0.54 | 1.55 | 0.000 | 0.016 | 0.371 | 12335 | tags=57%, list=22%, signal=73% |
| 27 | PID\_AR\_PATHWAY |  | 61 | 0.55 | 1.54 | 0.000 | 0.017 | 0.390 | 13960 | tags=57%, list=25%, signal=76% |
| 28 | PID\_ILK\_PATHWAY |  | 45 | 0.56 | 1.54 | 0.003 | 0.016 | 0.391 | 17888 | tags=67%, list=32%, signal=97% |
| 29 | PID\_VEGFR1\_2\_PATHWAY |  | 69 | 0.54 | 1.54 | 0.000 | 0.016 | 0.393 | 12355 | tags=51%, list=22%, signal=65% |
| 30 | PID\_WNT\_NONCANONICAL\_PATHWAY |  | 32 | 0.58 | 1.54 | 0.002 | 0.017 | 0.439 | 18619 | tags=72%, list=33%, signal=107% |
| 31 | PID\_HDAC\_CLASSII\_PATHWAY |  | 34 | 0.58 | 1.54 | 0.004 | 0.017 | 0.443 | 7162 | tags=47%, list=13%, signal=54% |
| 32 | PID\_PI3K\_PLC\_TRK\_PATHWAY |  | 36 | 0.57 | 1.53 | 0.003 | 0.018 | 0.486 | 8237 | tags=50%, list=15%, signal=58% |
| 33 | PID\_RB\_1PATHWAY |  | 65 | 0.54 | 1.53 | 0.000 | 0.018 | 0.494 | 14474 | tags=62%, list=26%, signal=83% |
| 34 | PID\_WNT\_CANONICAL\_PATHWAY |  | 20 | 0.62 | 1.52 | 0.007 | 0.018 | 0.510 | 10919 | tags=60%, list=19%, signal=74% |
| 35 | PID\_TNF\_PATHWAY |  | 46 | 0.55 | 1.52 | 0.001 | 0.019 | 0.529 | 17441 | tags=65%, list=31%, signal=94% |
| 36 | PID\_NCADHERIN\_PATHWAY |  | 33 | 0.57 | 1.52 | 0.007 | 0.019 | 0.536 | 15371 | tags=64%, list=27%, signal=87% |
| 37 | PID\_CDC42\_PATHWAY |  | 70 | 0.53 | 1.50 | 0.000 | 0.025 | 0.635 | 12355 | tags=54%, list=22%, signal=69% |
| 38 | PID\_A6B1\_A6B4\_INTEGRIN\_PATHWAY |  | 46 | 0.55 | 1.50 | 0.004 | 0.025 | 0.641 | 14907 | tags=57%, list=26%, signal=77% |
| 39 | PID\_BETA\_CATENIN\_DEG\_PATHWAY |  | 18 | 0.62 | 1.50 | 0.010 | 0.024 | 0.646 | 10919 | tags=61%, list=19%, signal=76% |
| 40 | PID\_INSULIN\_GLUCOSE\_PATHWAY |  | 26 | 0.59 | 1.49 | 0.010 | 0.025 | 0.662 | 12571 | tags=58%, list=22%, signal=74% |
| 41 | PID\_ECADHERIN\_NASCENT\_AJ\_PATHWAY |  | 39 | 0.55 | 1.49 | 0.004 | 0.024 | 0.664 | 12209 | tags=51%, list=22%, signal=65% |
| 42 | PID\_ERBB1\_INTERNALIZATION\_PATHWAY |  | 41 | 0.54 | 1.48 | 0.009 | 0.027 | 0.708 | 11602 | tags=51%, list=21%, signal=64% |
| 43 | PID\_PI3KCI\_AKT\_PATHWAY |  | 35 | 0.55 | 1.47 | 0.004 | 0.031 | 0.763 | 8781 | tags=49%, list=16%, signal=57% |
| 44 | PID\_NFAT\_3PATHWAY |  | 53 | 0.52 | 1.46 | 0.007 | 0.034 | 0.803 | 8787 | tags=45%, list=16%, signal=54% |
| 45 | PID\_ARF6\_TRAFFICKING\_PATHWAY |  | 49 | 0.52 | 1.46 | 0.012 | 0.033 | 0.804 | 14907 | tags=61%, list=26%, signal=83% |
| 46 | PID\_MET\_PATHWAY |  | 79 | 0.51 | 1.46 | 0.000 | 0.034 | 0.821 | 11056 | tags=48%, list=20%, signal=60% |
| 47 | PID\_FAK\_PATHWAY |  | 59 | 0.52 | 1.45 | 0.004 | 0.037 | 0.854 | 13691 | tags=58%, list=24%, signal=76% |
| 48 | PID\_RAC1\_PATHWAY |  | 54 | 0.51 | 1.44 | 0.009 | 0.040 | 0.881 | 11780 | tags=50%, list=21%, signal=63% |
| 49 | PID\_RETINOIC\_ACID\_PATHWAY |  | 30 | 0.55 | 1.44 | 0.021 | 0.040 | 0.882 | 20359 | tags=67%, list=36%, signal=104% |
| 50 | PID\_RAC1\_REG\_PATHWAY |  | 38 | 0.53 | 1.43 | 0.013 | 0.045 | 0.913 | 21575 | tags=71%, list=38%, signal=115% |
| 51 | PID\_SYNDECAN\_2\_PATHWAY |  | 33 | 0.54 | 1.43 | 0.023 | 0.045 | 0.917 | 15905 | tags=61%, list=28%, signal=84% |
| 52 | PID\_TGFBR\_PATHWAY |  | 54 | 0.52 | 1.43 | 0.007 | 0.044 | 0.918 | 17117 | tags=59%, list=30%, signal=85% |
| 53 | PID\_CDC42\_REG\_PATHWAY |  | 30 | 0.54 | 1.43 | 0.018 | 0.045 | 0.925 | 18521 | tags=67%, list=33%, signal=99% |
| 54 | PID\_ERBB1\_RECEPTOR\_PROXIMAL\_PATHWAY |  | 35 | 0.54 | 1.43 | 0.018 | 0.045 | 0.930 | 21490 | tags=80%, list=38%, signal=129% |
| 55 | PID\_S1P\_S1P1\_PATHWAY |  | 21 | 0.56 | 1.42 | 0.028 | 0.046 | 0.938 | 10368 | tags=57%, list=18%, signal=70% |
| 56 | PID\_EPHA2\_FWD\_PATHWAY |  | 19 | 0.58 | 1.42 | 0.030 | 0.047 | 0.940 | 10312 | tags=63%, list=18%, signal=77% |
| 57 | PID\_LIS1\_PATHWAY |  | 28 | 0.55 | 1.42 | 0.017 | 0.046 | 0.945 | 22850 | tags=82%, list=40%, signal=138% |
| 58 | PID\_VEGFR1\_PATHWAY |  | 26 | 0.55 | 1.42 | 0.023 | 0.046 | 0.947 | 16593 | tags=62%, list=29%, signal=87% |
| 59 | PID\_INSULIN\_PATHWAY |  | 44 | 0.52 | 1.42 | 0.006 | 0.046 | 0.949 | 17030 | tags=59%, list=30%, signal=85% |
| 60 | PID\_PRL\_SIGNALING\_EVENTS\_PATHWAY |  | 23 | 0.56 | 1.41 | 0.037 | 0.052 | 0.962 | 10312 | tags=61%, list=18%, signal=74% |
| 61 | PID\_PDGFRB\_PATHWAY |  | 129 | 0.48 | 1.40 | 0.001 | 0.054 | 0.967 | 12355 | tags=47%, list=22%, signal=60% |
| 62 | PID\_NECTIN\_PATHWAY |  | 30 | 0.53 | 1.40 | 0.032 | 0.055 | 0.967 | 13142 | tags=50%, list=23%, signal=65% |
| 63 | PID\_AVB3\_INTEGRIN\_PATHWAY |  | 74 | 0.49 | 1.39 | 0.009 | 0.063 | 0.988 | 10312 | tags=42%, list=18%, signal=51% |
| 64 | PID\_RHOA\_REG\_PATHWAY |  | 46 | 0.50 | 1.39 | 0.020 | 0.062 | 0.989 | 18521 | tags=65%, list=33%, signal=97% |
| 65 | PID\_PS1\_PATHWAY |  | 46 | 0.51 | 1.39 | 0.015 | 0.062 | 0.989 | 12782 | tags=61%, list=23%, signal=79% |
| 66 | PID\_ERBB1\_DOWNSTREAM\_PATHWAY |  | 105 | 0.47 | 1.37 | 0.002 | 0.073 | 0.996 | 12355 | tags=50%, list=22%, signal=64% |
| 67 | PID\_CERAMIDE\_PATHWAY |  | 44 | 0.50 | 1.37 | 0.028 | 0.074 | 0.997 | 17258 | tags=57%, list=31%, signal=82% |
| 68 | PID\_NOTCH\_PATHWAY |  | 59 | 0.49 | 1.37 | 0.017 | 0.077 | 0.999 | 12845 | tags=54%, list=23%, signal=70% |
| 69 | PID\_FOXO\_PATHWAY |  | 49 | 0.49 | 1.37 | 0.032 | 0.078 | 0.999 | 13133 | tags=49%, list=23%, signal=64% |
| 70 | PID\_TELOMERASE\_PATHWAY |  | 67 | 0.47 | 1.36 | 0.017 | 0.084 | 0.999 | 10621 | tags=45%, list=19%, signal=55% |
| 71 | PID\_CIRCADIAN\_PATHWAY |  | 15 | 0.58 | 1.36 | 0.062 | 0.084 | 0.999 | 11592 | tags=53%, list=21%, signal=67% |
| 72 | PID\_BETA\_CATENIN\_NUC\_PATHWAY |  | 80 | 0.47 | 1.35 | 0.009 | 0.093 | 0.999 | 15856 | tags=55%, list=28%, signal=76% |
| 73 | PID\_RAS\_PATHWAY |  | 30 | 0.52 | 1.34 | 0.046 | 0.097 | 1.000 | 14811 | tags=63%, list=26%, signal=86% |
| 74 | PID\_DELTA\_NP63\_PATHWAY |  | 47 | 0.48 | 1.34 | 0.033 | 0.098 | 1.000 | 14791 | tags=53%, list=26%, signal=72% |
| 75 | PID\_SYNDECAN\_1\_PATHWAY |  | 46 | 0.49 | 1.34 | 0.029 | 0.099 | 1.000 | 20731 | tags=65%, list=37%, signal=103% |
| 76 | PID\_TRKR\_PATHWAY |  | 61 | 0.47 | 1.34 | 0.021 | 0.099 | 1.000 | 8518 | tags=41%, list=15%, signal=48% |
| 77 | PID\_HIF2PATHWAY |  | 34 | 0.50 | 1.33 | 0.058 | 0.099 | 1.000 | 17123 | tags=65%, list=30%, signal=93% |
| 78 | PID\_TRAIL\_PATHWAY |  | 28 | 0.51 | 1.33 | 0.056 | 0.101 | 1.000 | 17805 | tags=61%, list=32%, signal=89% |
| 79 | PID\_RET\_PATHWAY |  | 39 | 0.50 | 1.33 | 0.046 | 0.102 | 1.000 | 8518 | tags=41%, list=15%, signal=48% |
| 80 | PID\_FAS\_PATHWAY |  | 38 | 0.49 | 1.33 | 0.049 | 0.101 | 1.000 | 17258 | tags=53%, list=31%, signal=76% |
| 81 | PID\_HIV\_NEF\_PATHWAY |  | 35 | 0.49 | 1.30 | 0.077 | 0.138 | 1.000 | 20276 | tags=57%, list=36%, signal=89% |
| 82 | PID\_ERB\_GENOMIC\_PATHWAY |  | 15 | 0.55 | 1.29 | 0.106 | 0.148 | 1.000 | 14537 | tags=73%, list=26%, signal=99% |
| 83 | PID\_SYNDECAN\_3\_PATHWAY |  | 17 | 0.53 | 1.29 | 0.102 | 0.147 | 1.000 | 10749 | tags=47%, list=19%, signal=58% |
| 84 | PID\_ERBB2\_ERBB3\_PATHWAY |  | 44 | 0.47 | 1.28 | 0.063 | 0.154 | 1.000 | 11980 | tags=50%, list=21%, signal=63% |
| 85 | PID\_ECADHERIN\_KERATINOCYTE\_PATHWAY |  | 21 | 0.51 | 1.27 | 0.103 | 0.167 | 1.000 | 10919 | tags=43%, list=19%, signal=53% |
| 86 | PID\_TAP63\_PATHWAY |  | 54 | 0.46 | 1.27 | 0.071 | 0.179 | 1.000 | 17334 | tags=56%, list=31%, signal=80% |
| 87 | PID\_P53\_DOWNSTREAM\_PATHWAY |  | 137 | 0.42 | 1.25 | 0.025 | 0.199 | 1.000 | 16117 | tags=53%, list=29%, signal=73% |
| 88 | PID\_LYMPH\_ANGIOGENESIS\_PATHWAY |  | 25 | 0.49 | 1.25 | 0.125 | 0.206 | 1.000 | 11634 | tags=44%, list=21%, signal=55% |
| 89 | PID\_NEPHRIN\_NEPH1\_PATHWAY |  | 31 | 0.47 | 1.23 | 0.139 | 0.228 | 1.000 | 17030 | tags=58%, list=30%, signal=83% |
| 90 | PID\_NETRIN\_PATHWAY |  | 32 | 0.46 | 1.23 | 0.144 | 0.244 | 1.000 | 17485 | tags=56%, list=31%, signal=81% |
| 91 | PID\_NFKAPPAB\_ATYPICAL\_PATHWAY |  | 17 | 0.51 | 1.22 | 0.185 | 0.249 | 1.000 | 14307 | tags=47%, list=25%, signal=63% |
| 92 | PID\_MAPK\_TRK\_PATHWAY |  | 34 | 0.46 | 1.22 | 0.147 | 0.253 | 1.000 | 12355 | tags=53%, list=22%, signal=68% |
| 93 | PID\_INTEGRIN\_A4B1\_PATHWAY |  | 33 | 0.46 | 1.20 | 0.176 | 0.286 | 1.000 | 19670 | tags=61%, list=35%, signal=93% |
| 94 | PID\_MYC\_REPRESS\_PATHWAY |  | 63 | 0.42 | 1.19 | 0.149 | 0.315 | 1.000 | 13804 | tags=49%, list=24%, signal=65% |
| 95 | PID\_EPO\_PATHWAY |  | 33 | 0.44 | 1.19 | 0.193 | 0.316 | 1.000 | 21310 | tags=58%, list=38%, signal=92% |
| 96 | PID\_IL8\_CXCR2\_PATHWAY |  | 34 | 0.45 | 1.19 | 0.180 | 0.313 | 1.000 | 8310 | tags=32%, list=15%, signal=38% |
| 97 | PID\_SMAD2\_3NUCLEAR\_PATHWAY |  | 82 | 0.41 | 1.18 | 0.120 | 0.328 | 1.000 | 14337 | tags=49%, list=25%, signal=65% |
| 98 | PID\_S1P\_S1P3\_PATHWAY |  | 29 | 0.44 | 1.18 | 0.223 | 0.325 | 1.000 | 15770 | tags=55%, list=28%, signal=76% |
| 99 | PID\_HDAC\_CLASSIII\_PATHWAY |  | 26 | 0.45 | 1.17 | 0.232 | 0.341 | 1.000 | 3967 | tags=31%, list=7%, signal=33% |
| 100 | PID\_ECADHERIN\_STABILIZATION\_PATHWAY |  | 41 | 0.43 | 1.17 | 0.207 | 0.340 | 1.000 | 12229 | tags=44%, list=22%, signal=56% |
| 101 | PID\_IL2\_PI3K\_PATHWAY |  | 34 | 0.44 | 1.17 | 0.233 | 0.342 | 1.000 | 14811 | tags=41%, list=26%, signal=56% |
| 102 | PID\_EPHA\_FWDPATHWAY |  | 34 | 0.44 | 1.16 | 0.237 | 0.358 | 1.000 | 7996 | tags=32%, list=14%, signal=38% |
| 103 | PID\_AR\_NONGENOMIC\_PATHWAY |  | 31 | 0.44 | 1.16 | 0.229 | 0.359 | 1.000 | 8518 | tags=45%, list=15%, signal=53% |
| 104 | PID\_CXCR3\_PATHWAY |  | 43 | 0.42 | 1.16 | 0.211 | 0.356 | 1.000 | 11056 | tags=42%, list=20%, signal=52% |
| 105 | PID\_RXR\_VDR\_PATHWAY |  | 26 | 0.45 | 1.16 | 0.259 | 0.353 | 1.000 | 13926 | tags=42%, list=25%, signal=56% |
| 106 | PID\_AJDISS\_2PATHWAY |  | 48 | 0.42 | 1.15 | 0.222 | 0.358 | 1.000 | 22165 | tags=60%, list=39%, signal=99% |
| 107 | PID\_TCPTP\_PATHWAY |  | 42 | 0.42 | 1.14 | 0.254 | 0.386 | 1.000 | 21310 | tags=64%, list=38%, signal=103% |
| 108 | PID\_INTEGRIN3\_PATHWAY |  | 43 | 0.42 | 1.14 | 0.246 | 0.384 | 1.000 | 10368 | tags=44%, list=18%, signal=54% |
| 109 | PID\_THROMBIN\_PAR1\_PATHWAY |  | 43 | 0.42 | 1.14 | 0.251 | 0.385 | 1.000 | 15295 | tags=51%, list=27%, signal=70% |
| 110 | PID\_REELIN\_PATHWAY |  | 28 | 0.43 | 1.13 | 0.284 | 0.403 | 1.000 | 12359 | tags=46%, list=22%, signal=59% |
| 111 | PID\_IL3\_PATHWAY |  | 26 | 0.44 | 1.12 | 0.317 | 0.427 | 1.000 | 10561 | tags=35%, list=19%, signal=43% |
| 112 | PID\_AR\_TF\_PATHWAY |  | 53 | 0.40 | 1.12 | 0.280 | 0.425 | 1.000 | 17123 | tags=53%, list=30%, signal=76% |
| 113 | PID\_INTEGRIN\_A9B1\_PATHWAY |  | 25 | 0.44 | 1.12 | 0.313 | 0.427 | 1.000 | 19927 | tags=64%, list=35%, signal=99% |
| 114 | PID\_CXCR4\_PATHWAY |  | 101 | 0.38 | 1.11 | 0.236 | 0.435 | 1.000 | 11667 | tags=40%, list=21%, signal=50% |
| 115 | PID\_P38\_ALPHA\_BETA\_DOWNSTREAM\_PATHWAY |  | 38 | 0.41 | 1.11 | 0.317 | 0.439 | 1.000 | 12640 | tags=42%, list=22%, signal=54% |
| 116 | PID\_RHOA\_PATHWAY |  | 45 | 0.40 | 1.11 | 0.313 | 0.438 | 1.000 | 17485 | tags=53%, list=31%, signal=77% |
| 117 | PID\_NFKAPPAB\_CANONICAL\_PATHWAY |  | 23 | 0.44 | 1.11 | 0.323 | 0.444 | 1.000 | 17184 | tags=48%, list=30%, signal=69% |
| 118 | PID\_KIT\_PATHWAY |  | 52 | 0.40 | 1.10 | 0.304 | 0.457 | 1.000 | 21310 | tags=52%, list=38%, signal=83% |
| 119 | PID\_ER\_NONGENOMIC\_PATHWAY |  | 40 | 0.40 | 1.09 | 0.346 | 0.478 | 1.000 | 15295 | tags=52%, list=27%, signal=72% |
| 120 | PID\_P75\_NTR\_PATHWAY |  | 68 | 0.38 | 1.09 | 0.329 | 0.478 | 1.000 | 14811 | tags=46%, list=26%, signal=62% |
| 121 | PID\_BMP\_PATHWAY |  | 42 | 0.39 | 1.09 | 0.358 | 0.477 | 1.000 | 16975 | tags=45%, list=30%, signal=65% |
| 122 | PID\_EPHB\_FWD\_PATHWAY |  | 40 | 0.40 | 1.09 | 0.348 | 0.477 | 1.000 | 13691 | tags=45%, list=24%, signal=59% |
| 123 | PID\_INTEGRIN\_CS\_PATHWAY |  | 26 | 0.42 | 1.08 | 0.351 | 0.480 | 1.000 | 22187 | tags=62%, list=39%, signal=101% |
| 124 | PID\_ERA\_GENOMIC\_PATHWAY |  | 64 | 0.38 | 1.08 | 0.326 | 0.494 | 1.000 | 18409 | tags=53%, list=33%, signal=79% |
| 125 | PID\_IL2\_STAT5\_PATHWAY |  | 30 | 0.40 | 1.07 | 0.403 | 0.501 | 1.000 | 19682 | tags=50%, list=35%, signal=77% |
| 126 | PID\_AVB3\_OPN\_PATHWAY |  | 31 | 0.41 | 1.07 | 0.394 | 0.501 | 1.000 | 20276 | tags=65%, list=36%, signal=101% |
| 127 | PID\_IL8\_CXCR1\_PATHWAY |  | 28 | 0.41 | 1.07 | 0.399 | 0.508 | 1.000 | 12578 | tags=36%, list=22%, signal=46% |
| 128 | PID\_CMYB\_PATHWAY |  | 84 | 0.36 | 1.05 | 0.376 | 0.535 | 1.000 | 17391 | tags=48%, list=31%, signal=69% |
| 129 | PID\_IFNG\_PATHWAY |  | 40 | 0.39 | 1.05 | 0.417 | 0.532 | 1.000 | 8768 | tags=30%, list=16%, signal=35% |
| 130 | PID\_LPA4\_PATHWAY |  | 15 | 0.44 | 1.05 | 0.433 | 0.536 | 1.000 | 24615 | tags=73%, list=44%, signal=130% |
| 131 | PID\_FGF\_PATHWAY |  | 55 | 0.37 | 1.04 | 0.435 | 0.549 | 1.000 | 17777 | tags=51%, list=31%, signal=74% |
| 132 | PID\_INTEGRIN1\_PATHWAY |  | 66 | 0.36 | 1.04 | 0.418 | 0.550 | 1.000 | 14924 | tags=50%, list=26%, signal=68% |
| 133 | PID\_TCR\_PATHWAY |  | 65 | 0.37 | 1.04 | 0.426 | 0.554 | 1.000 | 20276 | tags=45%, list=36%, signal=69% |
| 134 | PID\_CD8\_TCR\_PATHWAY |  | 53 | 0.37 | 1.03 | 0.433 | 0.562 | 1.000 | 23635 | tags=53%, list=42%, signal=91% |
| 135 | PID\_LYSOPHOSPHOLIPID\_PATHWAY |  | 65 | 0.36 | 1.03 | 0.434 | 0.572 | 1.000 | 15690 | tags=46%, list=28%, signal=64% |
| 136 | PID\_ALPHA\_SYNUCLEIN\_PATHWAY |  | 32 | 0.39 | 1.03 | 0.457 | 0.574 | 1.000 | 10646 | tags=31%, list=19%, signal=38% |
| 137 | PID\_HES\_HEY\_PATHWAY |  | 48 | 0.36 | 1.00 | 0.501 | 0.632 | 1.000 | 12782 | tags=40%, list=23%, signal=51% |
| 138 | PID\_TXA2PATHWAY |  | 56 | 0.35 | 0.99 | 0.540 | 0.640 | 1.000 | 8439 | tags=27%, list=15%, signal=31% |
| 139 | PID\_ALK1\_PATHWAY |  | 26 | 0.37 | 0.97 | 0.556 | 0.688 | 1.000 | 15350 | tags=42%, list=27%, signal=58% |
| 140 | PID\_RHODOPSIN\_PATHWAY |  | 23 | 0.38 | 0.96 | 0.582 | 0.700 | 1.000 | 14835 | tags=48%, list=26%, signal=65% |
| 141 | PID\_P38\_MKK3\_6PATHWAY |  | 26 | 0.38 | 0.96 | 0.591 | 0.714 | 1.000 | 17069 | tags=54%, list=30%, signal=77% |
| 142 | PID\_SYNDECAN\_4\_PATHWAY |  | 32 | 0.36 | 0.95 | 0.616 | 0.729 | 1.000 | 15770 | tags=50%, list=28%, signal=69% |
| 143 | PID\_ERBB\_NETWORK\_PATHWAY |  | 15 | 0.40 | 0.94 | 0.575 | 0.729 | 1.000 | 5054 | tags=20%, list=9%, signal=22% |
| 144 | PID\_ENDOTHELIN\_PATHWAY |  | 63 | 0.34 | 0.94 | 0.645 | 0.730 | 1.000 | 17485 | tags=46%, list=31%, signal=67% |
| 145 | PID\_CONE\_PATHWAY |  | 23 | 0.37 | 0.94 | 0.608 | 0.737 | 1.000 | 15198 | tags=43%, list=27%, signal=59% |
| 146 | PID\_FCER1\_PATHWAY |  | 60 | 0.33 | 0.93 | 0.667 | 0.745 | 1.000 | 14331 | tags=37%, list=25%, signal=49% |
| 147 | PID\_IL2\_1PATHWAY |  | 55 | 0.33 | 0.92 | 0.686 | 0.768 | 1.000 | 5800 | tags=24%, list=10%, signal=26% |
| 148 | PID\_IL1\_PATHWAY |  | 34 | 0.34 | 0.91 | 0.669 | 0.775 | 1.000 | 14811 | tags=44%, list=26%, signal=60% |
| 149 | PID\_PDGFRA\_PATHWAY |  | 22 | 0.36 | 0.90 | 0.657 | 0.787 | 1.000 | 10785 | tags=36%, list=19%, signal=45% |
| 150 | PID\_IL4\_2PATHWAY |  | 64 | 0.32 | 0.90 | 0.722 | 0.787 | 1.000 | 24446 | tags=58%, list=43%, signal=102% |
| 151 | PID\_PTP1B\_PATHWAY |  | 52 | 0.32 | 0.90 | 0.722 | 0.786 | 1.000 | 5831 | tags=21%, list=10%, signal=24% |
| 152 | PID\_HEDGEHOG\_2PATHWAY |  | 22 | 0.36 | 0.89 | 0.679 | 0.787 | 1.000 | 13883 | tags=41%, list=25%, signal=54% |
| 153 | PID\_GMCSF\_PATHWAY |  | 36 | 0.33 | 0.88 | 0.708 | 0.793 | 1.000 | 5666 | tags=22%, list=10%, signal=25% |
| 154 | PID\_CD40\_PATHWAY |  | 31 | 0.33 | 0.88 | 0.711 | 0.801 | 1.000 | 8781 | tags=26%, list=16%, signal=31% |
| 155 | PID\_WNT\_SIGNALING\_PATHWAY |  | 28 | 0.33 | 0.86 | 0.737 | 0.824 | 1.000 | 25559 | tags=61%, list=45%, signal=111% |
| 156 | PID\_FRA\_PATHWAY |  | 37 | 0.31 | 0.84 | 0.775 | 0.850 | 1.000 | 9315 | tags=30%, list=16%, signal=36% |
| 157 | PID\_REG\_GR\_PATHWAY |  | 82 | 0.29 | 0.84 | 0.831 | 0.849 | 1.000 | 18352 | tags=43%, list=32%, signal=63% |
| 158 | PID\_INTEGRIN5\_PATHWAY |  | 17 | 0.35 | 0.83 | 0.767 | 0.846 | 1.000 | 9818 | tags=29%, list=17%, signal=36% |
| 159 | PID\_BCR\_5PATHWAY |  | 63 | 0.29 | 0.81 | 0.848 | 0.875 | 1.000 | 20526 | tags=43%, list=36%, signal=67% |
| 160 | PID\_SHP2\_PATHWAY |  | 57 | 0.29 | 0.81 | 0.861 | 0.873 | 1.000 | 21533 | tags=49%, list=38%, signal=79% |
| 161 | PID\_ARF6\_PATHWAY |  | 35 | 0.29 | 0.78 | 0.847 | 0.898 | 1.000 | 6669 | tags=20%, list=12%, signal=23% |
| 162 | PID\_ANGIOPOIETIN\_RECEPTOR\_PATHWAY |  | 49 | 0.28 | 0.77 | 0.863 | 0.898 | 1.000 | 17875 | tags=43%, list=32%, signal=63% |
| 163 | PID\_S1P\_S1P2\_PATHWAY |  | 24 | 0.29 | 0.75 | 0.864 | 0.918 | 1.000 | 15295 | tags=46%, list=27%, signal=63% |
| 164 | PID\_CD8\_TCR\_DOWNSTREAM\_PATHWAY |  | 65 | 0.26 | 0.73 | 0.930 | 0.928 | 1.000 | 14031 | tags=26%, list=25%, signal=35% |
| 165 | PID\_ERBB4\_PATHWAY |  | 38 | 0.26 | 0.70 | 0.931 | 0.947 | 1.000 | 18468 | tags=42%, list=33%, signal=62% |
| 166 | PID\_S1P\_META\_PATHWAY |  | 21 | 0.26 | 0.65 | 0.933 | 0.971 | 1.000 | 22165 | tags=57%, list=39%, signal=94% |
| 167 | PID\_TOLL\_ENDOGENOUS\_PATHWAY |  | 24 | 0.26 | 0.65 | 0.947 | 0.966 | 1.000 | 16088 | tags=38%, list=28%, signal=52% |
| 168 | PID\_GLYPICAN\_1PATHWAY |  | 27 | 0.25 | 0.64 | 0.942 | 0.963 | 1.000 | 13883 | tags=26%, list=25%, signal=34% |
| 169 | PID\_THROMBIN\_PAR4\_PATHWAY |  | 15 | 0.26 | 0.61 | 0.948 | 0.970 | 1.000 | 14216 | tags=33%, list=25%, signal=45% |
Table: Gene sets enriched in phenotype **1 (47 samples)**[plain text format]****

  
